# Patient-reported outcomes after primary or revision total hip arthroplasty: A propensity score-matched Asian cohort study

**DOI:** 10.1371/journal.pone.0252112

**Published:** 2021-05-27

**Authors:** Satoru Harada, Satoshi Hamai, Kyohei Shiomoto, Daisuke Hara, Masanori Fujii, Satoshi Ikemura, Goro Motomura, Yasuharu Nakashima

**Affiliations:** 1 Department of Orthopaedic Surgery, Faculty of Medical Sciences, Kyushu University, Fukuoka, Japan; 2 Department of Medical-Engineering Collaboration for Healthy Longevity, Kyushu University, Fukuoka, Japan; Assiut University Faculty of Medicine, EGYPT

## Abstract

**Background:**

Few studies have compared patient-reported outcome measures (PROMs) between primary and revision total hip arthroplasty (THA). We investigated and compared PROMs between propensity score-matched primary and revision THA in an Asian cohort.

**Methods:**

The Oxford Hip Score (OHS) and University of California-Los Angeles (UCLA) activity score, satisfaction score, and Short Form-12 Health Survey (SF-12) were compared between 110 primary and 110 revision THAs after propensity score matching. Multivariate analyses were performed to determine which factors, including patients’ demographics, indication for revision, and pre-operative PROMs, were associated with post-operative PROMs in the revision THA cohort.

**Results:**

The revision THA cohort demonstrated significantly lower post-operative OHS, UCLA activity score, and satisfaction score (10% decrease on average) than those in the primary THA cohort *(P <* .*05)*. The difference in SF-12 mental component summary measure (MCS) between the two cohorts was statistically insignificant (*P* = .24). In multivariate analysis for the revision THA cohort, lower post-operative UCLA activity score was significantly associated with higher BMI and lower pre-operative UCLA activity score (*P* < .05).

**Conclusion:**

Revision THA was associated with a modest but significant decrease in physical PROMs as compared with primary THA. Pre-operative UCLA activity score significantly affected the post-operative physical outcome measures in the revision THA cohort. However, post-operative SF-12 MCS was comparable between the primary and revision THA cohorts.

## Introduction

Total hip arthroplasty (THA) is an effective treatment of end-stage hip osteoarthritis (OA) to restore patients’ quality of life (QOL) [1]). Despite the widely recognized success of the THA [2], the incidence of revision THA is on the rise. Aseptic loosening, recurrent dislocation, infection, or periprosthetic fracture [3–5] are the primary reasons attributed to this increased rate of revision THA. For primary THA, the 10-year and 20-year implant survival rates have been reported as 95.6% and 85%, respectively [6]. The frequency of revision THA is projected to double by 2026 in the United States [7–9]. In comparison with primary THA, revision THA is associated with more short- and long-term complications and higher mortality rates [10, 11].

Patients report high expectations for improvements in pain, function, and QOL even after revision surgery [12]. The use of patient-reported outcome measures (PROMs) to evaluate the clinical effect of arthroplasty procedures yields unique insight into the patients’ actual and perceived physical benefits of revision THA. Although several studies of post-operative outcomes of primary THA using PROMs have been reported [13–16], the comparison of PROMs between revision and primary THA using propensity score matching has not been evaluated [17, 18]. Propensity score matching minimizes the effects of patient-specific factors including age, sex, BMI, and follow-up duration leading to a more accurate characterization of the effects of surgical procedures [19].

The primary objective of this study was to evaluate the PROMs of revision THA at mid- to long-term follow-up in an Asian cohort. The secondary objectives were to compare the PROMs of the revision and primary THA cohorts matched based on the propensity score, and to determine the factors influencing PROMs in revision THA.

## Materials and methods

### Revision and primary THA cohorts

**Fig 1:**
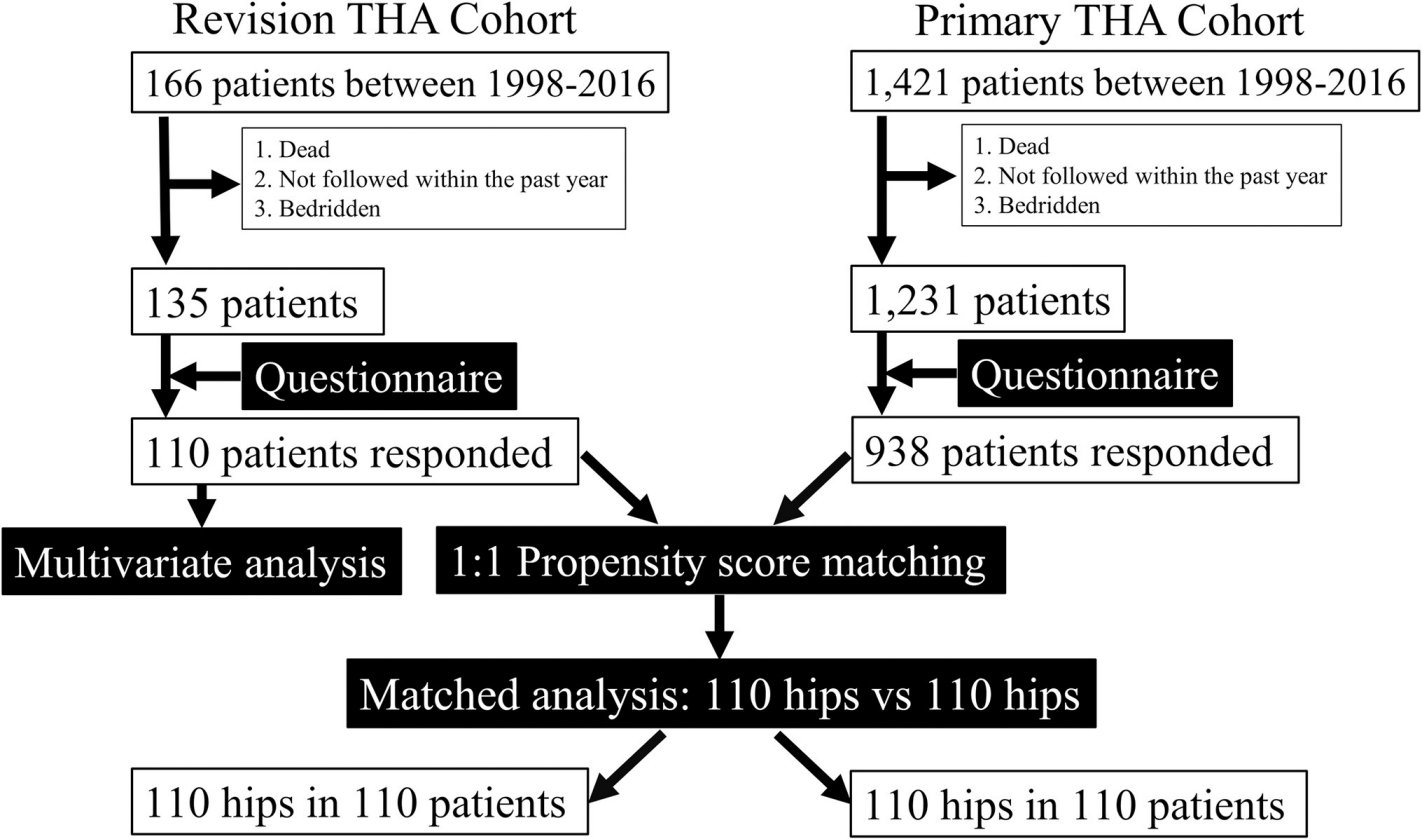
Schematic representation of the study cohort inclusion process and study design.

This study was approved by the Committee of Ethics in Kyushu University (IRB number 30–91) and performed in accordance with the Ethical Standards of the Helsinki Declaration. We retrospectively reviewed the data for 166 patients who underwent revision THA and 1421 patients who underwent primary THA between January 1998 and December 2016. Among these revision and primary THA patients, 135 and 1231 of them satisfied all the following inclusion criteria, respectively: (1) alive at the time of the survey, (2) at least a year elapsed since last surgery, (3) evaluation by a surgeon within the past one year, and (4) absence of any physical or mental disorders unrelated to the hip joint that may lead to bed rest. The exclusion criteria included insufficient responses in the questionnaire. The survey questionnaire was mailed to all patients, of which 110 revision THA patients (120 hips, 81%) and 938 primary THA patients (1031 hips, 76.2%) completed and returned the questionnaire with written informed consent (Fig 1). Data were handled in accordance with the Ethical Standards of the Helsinki Declaration.

### Questionnaire

The survey questionnaire collected information on: (1) all patient-derived scores, including the post-operative Japanese Oxford Hip Score (OHS) [20–22], (2) patients’ pre- and post-operative physical activity levels determined using the University of California-Los Angeles (UCLA) activity scale score [17, 23, 24], (3) post-operative Short Form-12 Health Survey (SF-12) [25–27], and (4) post-operative satisfaction score [28]. The OHS and UCLA activity scores are validated, reliable, and self-reported metrics for patients with hip OA [23, 29, 30]. The OHS, a disease-specific QOL assessment, is a 12-item measure of patient-reported outcomes developed to assess function and pain in THA patients. Each item’s score is on a scale of 0 to 4, and the total score ranges from 0 to 48 with higher scores indicating better outcomes. The UCLA activity score measures physical activity levels, and the total score ranges from 1 to 10 with higher scores indicating a higher level of physical activity. The SF-12 is a generic and well-established health-related QOL measure and is comprised of a subset of 12 items from the SF-36 scale. Information from all 12 items is used to construct physical, mental, and role/social component summary measures (PCS, MCS, and RCS) [26]. Satisfaction score consists of a 4-point Likert scale with response categories consisting of very satisfied (100), somewhat satisfied (75), somewhat dissatisfied (50), and very dissatisfied (25); an average score of four items was used in this study [28]. All demographic factors, including age at the time of surgery, sex, body mass index (BMI), follow-up duration, and indication for primary and revision surgeries were obtained from the patients’ medical records.

### Statistical analysis

First, propensity score matching was performed between 110 revision THA patients and 938 primary THA patients (Fig 1). Based on the propensity score, 1:1 matched cohorts were generated to facilitate comparison between revision and primary THA patients [18, 31, 32]. Age at the time of surgery, sex, BMI, and follow-up duration were included as confounders when calculating the propensity score by multivariate logistic regression analysis for each patient. The patients were matched using the nearest neighbor technique, with a predefined caliper width equal to 0.2 of the standard deviation of the logit of the propensity score [32]. The propensity score matching resulted in a successful match for all variables (Table 1). Consequently, 110 patients (110 hips) were included for the propensity score-matched analysis in each cohort—revision and primary THA (Fig 1). Wilcoxon signed-rank test and the chi-square test were used, as appropriate, to compare the characteristics between the revision and primary THA cohorts.

**Table 1:** Patients demographics of propensity score-matched cohorts.

Parameters	Revision THA (N = 110)	Primary THA (N = 120)	P value^a^
Age at surgery, years	65.2 ± 10.1 (39–85)	64.8 ± 10.3 (40–86)	0.64
Gender (male; female), hips	34; 76	36; 84	0.77
Body mass index, kg/m^2^	22.8 ± 3.2 (16.1–29.2)	23.0 ± 3.0 (16.1–29.2)	0.96
Follow-up duration, years	12.1 ± 4.0 (3.7–19.2)	12.0 ± 3.7 (3.7–19.3)	0.46
Indication for initial THA (OA; ONFH; RA; fracture), hips	92; 13; 2; 3	98; 18; 3; 0	0.23
Initial surgery facility (this institution; other institution), hips	75; 35	120; 0	N/A
Period from initial surgery to revision, years	13.8 ± 7.4 (0.1–30.4)	N/A	N/A
Number of revisions (one; two; three)	99; 10; 1	N/A	N/A

THA: total hip arthroplasty, OA: osteoarthritis, ONFH: osteonecrosis of the femoral head, RA: rheumatoid arthritis.

Continuous values are expressed as mean ± standard deviation (range).

Second, Wilcoxon signed-rank test was used to compare the pre-and post-operative UCLA activity scores, while the chi-square test was used to compare the post-operative OHS score, patient satisfaction, PCS, MCS, and RCS in revision and primary THA cohorts. To identify factors influencing PROMs in revision THA cohorts, multivariate logistic regression analysis was performed using the following factors: patients’ demographics (age, sex, BMI, follow-up duration, and time from initial surgery to revision surgery), pre-operative UCLA activity score, and the indication for revision (excessive wear of conventional polyethylene [CPE], aseptic loosening, recurrent dislocation, infection, periprosthetic fracture, and broken prosthesis).

Values are expressed as mean ± standard deviation. The significance level was set at < .05 for all tests. All statistical analyses were performed using JMP Pro Version 14.0 (SAS Institute Inc, Cary, NC).

## Results

### Patients’ demographics in the primary THA cohort

The primary THA cohort included 76 females and 34 males after 1:1 propensity score matching. The average age at the time of surgery, BMI, and follow-up duration were 64.3 ± 10.4 years (range: 40–86), 23.0 ± 3.0 kg/m^2^ (range: 16.1–29.6), and 12.0 ± 3.7 years (range: 3.7–19.3), respectively. The primary THA patient characteristics are presented in Table 1. The average age at the time of surgery, sex, BMI, follow-up duration, and indication for initial THA in the two cohorts were similar (*P* = .85, .77, .96, .87, and .23, respectively; Table 1).

### Patients’ demographics in the revision THA cohort

The revision THA cohort included 76 females and 34 males. The mean age at the time of surgery, BMI, and follow-up duration were 64.8 ± 10.3 years (range: 39–85), 23.0 ± 3.2 kg/m^2^ (range: 16.1–29.2), and 12.1± 3.9 years (range: 3.7–18.2), respectively. The mean period from the initial surgery to revision surgery was 13.8 years (range: 0.1–30.4), and the revision surgery was performed once in 99 hips, twice in 10 hips, and thrice in 1 hip. The revision THA patient characteristics are presented in Table 1. The indication for revision THA was excessive wear of CPE without loosening in 52 hips (43%), aseptic loosening in 45 hips (37%), recurrent dislocation in 10 hips (98), and infection in 9 hips (7.5%) (Table 2).

**Table 2:** Indications for revision.

Indication for Revision	Hips (Percentage)
Excessive CPE wear	52 (43.7%)
Aseptic loosening	45 (37.8%)
Acetabular component	16(13.4%)
Femoral component	13 (10.9%)
Both components	16 (13.4%)
Frequent dislocation	10 (8.4%)
Infection	9 (7.6%)
Periprosthetic fracture	2 (1.7%)
Mechanical failure of implants	2 (1.7%)

CPE; conventional polyethylene, the group included in the excessive CPE wear did not have loosening and were treated with liner and head exchange.

### PROMs in the primary THA cohort

The average post-operative OHS score was 40.8 ± 8.2 (range: 20–48). The average UCLA activity score increased from 4.3 ± 1.9 (range: 1–10) pre-operatively to 5.2 ± 2.0 (range: 1–10) post-operatively (*P* < .01). The post-operative average PCS, MCS, and RCS were 44.8 ± 13.0 (range: 28.1–64.1), 55.7 ± 7.1 (range: 38.3–70.9), and 46.0± 13.5 (range: 25.3–60.4), respectively. The average post-operative satisfaction score was 85.2 ± 20.2 (range: 25–100) (Table 3).

**Table 3:** Summary of patient-reported outcomes.

Parameters	Revision THA (N = 120)	Primary THA (N = 120)	P value^a^
OHS	38.3 ± 8.7	40.6 ± 8.2	< .01
	(10–48)	(20–48)	
Pre-operative UCLA activity score	3.7 ± 1.6	4.2 ± 1.5	< .01
	(1–10)	(1–10)	
Post-operative UCLA activity score	4.4 ± 1.5	5.2 ± 2.0	< .01
	(1–10)	(1–10)	
Patient satisfaction	82.9 ± 18.9	85.2 ± 20.2	< .01
	(31.25–100)	(25–100)	
SF-12 PCS	41.3 ± 15.9	41.8 ± 14.3	0.93
	(6.5–65.1)	(28.1–64.1)	
SF-12 MCS	56.2 ± 9.9	56.8 ± 8.6	0.56
	(38.1–74.7)	(38.3–70.9)	
SF-12 RCS	45.0 ± 11.5	46.5 ± 10.2	0.36
	(13.2–70.6)	(25.3–60.4)	

THA, total hip arthroplasty; OHS, Oxford Hip Score; UCLA, University of California, Los Angeles; SF-12, Short Form-12 Health Survey.

Continuous values are expressed as mean ± standard deviation (range).

### PROMs in the revision THA cohort

The average post-operative OHS score was 38.0 ± 8.7 (range: 10–48). The average UCLA activity score increased from 3.5 ± 1.4 (range: 1–10) pre-operatively to 4.3 ± 1.9 (range: 1–10) post-operatively (*P* < .01). The post-operative average PCS, MCS, and RCS were 45.7 ± 10.4 (range: 6.5–69.5), 57.7 ± 8.1 (range: 38.1–74.7), and 44.8 ± 9.7 (range: 13.2–70.6), respectively. The average post-operative satisfaction score was 82.5 ± 19.6 (range: 25–100) (Table 3). The effect of the underlying reason for revision THA on PROMS is shown in Table 4. The post-operative PCS in the infection subcohort (29.0) was significantly lower (*P* < .05) than the PCS in the excessive CPE wear (45.4), aseptic loosening (47.6), and recurrent dislocation (50.7) subcohorts. The post-operative PCS and RCS were significantly lower in the two revision (re-revision) surgeries subcohort than in the one revision surgery subcohort (47.1 vs. 36.3 and 46.1 vs. 38.3, respectively) (*P* < .05).

**Table 4:** Comparison of the patient-reported outcomes by reasons of revision THA.

Parameters	Indication for revision THA				P value
	Excessive CPE wear (N = 61)	Aseptic loosening (N = 46)	Multiple dislocation (N = 10)	Infection (N = 11)	
OHS	38.9 ± 8.8	38.0 ± 8.0	40.6 ± 9.1	38.3 ± 7.4	0.84
UCLA activity score	4.5 ± 1.5	4.1 ± 1.4	5.6 ± 1.6	5.1 ± 1.4	0.11
Satisfaction	82.9 ± 17.8	81.7 ± 16.2	83.8 ± 27.5	83.0 ± 26.4	0.91
SF-12 PCS	46.2 ± 12.2	32.0 ± 19.1	43.6 ± 14.8	50.2 ± 3.5	0.09
SF-12 MCS	55.3 ± 9.8	54.0 ± 9.8	64.6 ± 1.7	69.2 ± 2.4	0.12
SF-12 RCS	43.9 ± 43.8	43.9 ± 15.9	53.8 ± 4.2	48.9 ± 1.0	0.23
	Indication for revision THA				
	Aseptic loosening				
	cup (N = 17)	cup, stem (N = 16)	stem (N = 13)		
OHS	37.5 ± 7.9	37.5 ± 7.5	39.3 ± 9.2		0.81
UCLA activity score	4.0 ± 8.9	4.0 ± 1.4	4.3 ± 1.9		0.78
Satisfaction	84 ± 12.9	83.1 ± 13.5	77.9 ± 21.9		0.62
SF-12 PCS	34.2 ± 18.4	37.9 ± 6.7	25.7 ± 25.9		0.73
SF-12 MCS	54.8 ± 8.0	66.3 ± 11.9	46.6 ± 4.9		0.08
SF-12 RCS	46.1 ± 25.5	46.9 ± 16.8	46.9 ± 16.8		0.50
	Indication for initial surgery in revision THA cohort				
	OA (N = 106)	ON (N = 13)	RA (N = 2)	Fracture (N = 3)	
OHS	39.0 ± 8.3	37.2 ± 8.5	45.0 ± 1.4	40.3 ± 10.8	0.64
UCLA activity score	4.3 ± 1.4	4.6 ± 1.5	7.5 ± 0.7	6.0 ± 2.6	0.12
Satisfaction	83.9 ± 18.0	75.3 ± 25.1	90.0 ± 14.1	87.5 ± 17.7	0.44
SF-12 PCS	41.3 ± 17.6	38.7 ± 9.9	41.0 ± 13.0	52.6	0.91
SF-12 MCS	54.8 ± 10.3	60.8 ± 8.8	62.3 ± 7.1	67.6	0.38
SF-12 RCS	45.6 ± 11.8	37.5 ± 12.1	47.2 ± 16.8	48.2	0.71
	Number of revisions				
	One (N = 107)	Two (N = 12)	Three (N = 1)		
OHS	39.1 ± 8.3	37.3 ± 7.8	44		0.63
UCLA activity score	4.4 ± 1.4	4.7 ± 1.6	2		0.21
Satisfaction	83.1 ± 17.9	81.9 ± 24.8	75		0.89
SF-12 PCS	42.2 ± 16.2	29.5 ± 1.1	30.6		0.46
SF-12 MCS	56.6 ± 10.1	51.7 ± 4.6	47.1		0.55
SF-12 RCS	45.6 ± 11.7	37.3	36		0.48

THA, total hip arthroplasty; BMI, body mass index; CPE, conventional polyethylene; OHS, Oxford Hip Score; UCLA, University of California, Los Angeles; PCS, physical component summary; MCS, mental component summary; RCS, role/social component summary; OA, osteoarthritis; ON, osteonecrosis; RA, rheumatoid arthritis.

^a^ *P* < .05 for the comparison of PROMs between excessive CPE wear and infection cohorts.

^b^ *P* < .05 for the comparison of PROMs between aseptic loosening wear and infection cohorts.

^c^ *P* < .05 for the comparison of PROMs between aseptic recurrent dislocation and infection cohorts.

^d^ *P* < .05 for the comparison of PROMs between one and two revision surgeries cohorts.

The variables without small letters (^a, b, c, d^) mean the differences to be insignificant (*P >* .*05*).

### Revision vs primary THA after propensity score matching

In the matched analysis, primary THA was significantly better than revision THA in post-operative OHS (40.8 vs. 38.0), pre- and post-operative UCLA activity score (4.3 vs.3.5 and 5.2 vs. 4.3, respectively), and post-operative satisfaction score (85.2 vs. 82.5) (*P* < .01) (Table 3). The UCLA activity score increased post-operatively by 0.8 ± 1.7 (range: -5–6) for revision THA and 0.9 ± 2.1 (range: -7–7) for primary THA (*P* = .54). Post-operative OHS, post-operative UCLA activity score, and post-operative satisfaction in the revision THA cohort were 93%, 83%, and 96% (average: 90%) of the corresponding metrics in the primary THA cohort. SF-12 PCS, MCS, and RCS were comparable in the revision and primary THA cohorts (*P* = .98, .24, and .97, respectively; Table 3).

Multivariate analysis failed to identify factors correlated with post-operative OHS (Table 5). Higher BMI and lower pre-operative UCLA activity scores were negatively associated with post-operative UCLA activity score (*P* < .01 and < .01, respectively). Lower pre-operative UCLA activity score and mechanical failure of implants (two hips with broken stem) were negatively associated with post-operative satisfaction score (*P* = .04 and .02, respectively). Lower pre-operative UCLA activity scores were negatively associated with SF-12 PCS and RCS (*P* = .02 and .04, respectively). Additionally, higher age at surgery and infection were negatively associated with SF-12 MCS (*P* < .01 and = .01, respectively).

**Table 5:** Factors influencing PROMs in revision THA.

Factors	OHS	UCLA activity score	Satisfaction	SF-12 PCS	SF-12 MCS	SF-12 RCS
Demographic data						
Age at surgery	.74	.66	.74	.44	.03*	.40
Sex (Female)	.64	.09	.48	.66	.80	.89
BMI	.84	.01*	.72	.12	.12	.39
Follow-up duration	.54	.44	.52	.29	.27	.90
Period from initial surgery to revision	.57	.49	.32	.25	.40	.90
Indication for revision						
Excessive CPE wear	.84	.50	.51	.37	.77	.51
Aseptic loosening	.77	.64	.56	.28	.77	.34
Frequent dislocation	.70	.12	.96	N/A	N/A	N/A
Infection	.55	.29	.98	.81	< .01*	.28
Periprosthetic fracture	.94	.87	.81	N/A	N/A	N/A
Prosthesis broken	.87	.93	.04*	N/A	N/A	N/A
Pre-operative UCLA activity score	.31	< .01*	.03*	.02*	.88	.04*

THA, total hip arthroplasty; BMI, body mass index; CPE, conventional polyethylene; OHS, Oxford Hip score; UCLA, University of California, Los Angeles; SF-12, Short Form-12 Health Survey; PCS, physical component summary; MCS, mental component summary; RCS, role/social component summary.

* Statistically significant (*P* < .05).

## Discussion

The majority of patients with either primary or revision THA achieved good to excellent clinical results 12 years on average after the operation, with equivalent improvement of UCLA activity score. Nevertheless, there was a modest (10%) but statistically significant decrease in the physical outcome measures (post-operative OHS and UCLA activity score) and satisfaction score following revision THA than those after primary THA. The physical outcomes and satisfaction were affected by the pre-operative UCLA activity score. The mental QOL, measured via SF-12 MCS, was comparable between primary and revision THA.

Few previous studies have addressed the differences in clinical outcomes between primary and revision THA; however, these studies were characterized by certain important methodological drawbacks [12, 33, 34]. Lübbeke et al. assessed the patient status five years post-operatively without comparing the results to pre-operative PROMs [12]. The cohorts were not matched for age, sex, BMI, and follow-up duration which may influence PROMs. Patil et al. obtained the Western Ontario and McMaster Universities Osteoarthritis Index (WOMAC) scores pre-operatively as well as 1–3 years post-operatively in patients who underwent revision THA, compared to primary THA [33]. However, their cohorts were not specifically matched for potential confounders. Espehaug et al. reported the comparison of PROMs for revision THA cohort with primary THA cohort; however, the follow-up duration was substantially different after primary THA (5.2 years) and revision THA (2.3 years) [34]. The strengths of the present study include characterization of a large revision THA cohort with mid- to long-term follow-up. Furthermore, we compared the revision THA cohort with the primary THA cohort using propensity score matching for age at surgery, sex, BMI, and follow-up duration.

In this study, the average post-operative OHS, UCLA activity score, and satisfaction were significantly different between the revision THA (40.8, 5.2, and 85.2, respectively) and the primary THA (40.8, 5.2, and 85.2, respectively) cohorts. Evaluation of physical outcome measures revealed that OHS and UCLA activity scores in the revision THA were lower than in the primary THA. These findings are in agreement with several previous reports [33–37]. Weber et al. reported that revision arthroplasty is associated with poorer physical outcomes compared to the primary surgery [35]. The patient satisfaction score was also reported to be better in the primary THA cohort than in the revision THA cohort [35]. Overall, these findings suggest that the cases of revision THA pose a major challenge for orthopedists [5, 7]. The average UCLA activity score increased from 3.5 pre-operatively to 4.3 post-operatively in the revision THA cohort. Consistent with this, Postler et al. also reported an improvement in UCLA activity score (4.1 vs. 5.2) after revision surgery at 4-years follow-up [38]. In contrast with the physical outcomes, the mental outcomes measured using the SF-12 MCS did not differ significantly between primary and revision THA cohorts. Although a pessimistic outlook has been reported to influence PROMs [39, 40], the present study did not reveal a correlation between mental characteristics and post-operative clinical outcomes after THA.

In the present study, the post-operative PCS in patients who underwent revision due to infection was significantly lower than the PCS in those with excessive CPE wear, aseptic loosening, and recurrent dislocation as indications for revision. Herman et al. also reported that PROMs (SF12- PCS, Harris Hip Score, and WOMAC) for the infection cohort were significantly worse than the noninfected controls [41]. Other previous studies have shown poor PROMs for revision THA due to dislocation or periprosthetic fracture and infection [41, 42]. Turnbull et al. reported that revision THA for aseptic loosening was associated with better PROMs (OHS and UCLA activity score) at 7-year follow-up, while revision for periprosthetic fracture had the worst PROMs and recurrent dislocation was associated with lower patient satisfaction [42]. There are no previous reports comparing PROMs between revision and re-revision THA [43]. Both post-operative PCS and RCS were significantly lower in the re-revision subcohort than in the revision cohort in the present study.

Multivariate analysis showed a negative association between higher BMI and post-operative UCLA activity score and between higher age at surgery and SF-12 MCS. A similar trend for BMI and UCLA activity score was observed in the revision THA cohort in previous studies [12, 17]. Lübbecke et al. showed that older age and obesity can at least partially explain functional outcomes after revision THA [12]. Hara et al. reported an association of lower post-operative UCLA activity scores with higher BMI in primary THA [17]. In this study, lower pre-operative UCLA activity score was negatively associated with post-operative UCLA activity score, satisfaction score, and SF-12 PCS as well as RCS. It is well established that pre-operative functional capacity predicts post-operative clinical outcomes in primary THA [37, 44]. However, few studies have examined this association in revision THA. Turnbull et al. demonstrated the predictive correlation between pre- and post-operative UCLA activity scores in revision THA at 7-year follow-up [42]. Our study showed similar results at an even longer 12-year follow-up. As unrealistic patient expectations are associated with unsatisfactory post-operative outcomes [45, 46], it is necessary to inform patients before revision THA about the realistic levels of the expected achievement in post-operative function, activity, and QOL: generally 10% decrease in the OHS, UCLA activity score and satisfaction score, but equivalent SF-12 scores compared to the primary THA. The observation that the levels of pre-operative activity are largely related to physical outcomes after revision THA at mid- to long-term follow-up can guide surgeons in counseling patients about the predicted functional outcomes after revision surgery. In terms of subtypes and number of revision, infection negatively influenced post-operative SF-12 MCS and re-revision negatively influenced both PCS and RCS.

There are certain important limitations to our study. First, the study design is a retrospective analysis making it susceptible to potential bias. Therefore, we attempted to reduce this bias and matched the cohorts in terms of age, sex, BMI, and follow-up duration using the propensity score matching methodology. A further prospective study with a longer follow-up duration to examine the factors influencing post-operative PROMs for revision THA is needed. Second, not all patients were accounted for due to unreturned questionnaires or incomplete data in the questionnaires returned. Notably, the response rate with sufficient data in this study was 81.4%, which is higher than in previously reported studies [47–49].

## Conclusion

The revision THA was associated with a significant decrease (approximately 10%) in physical outcome measures (OHS and UCLA activity score) and satisfaction score compared with propensity score-matched primary THA. The physical outcome measures and satisfaction score were affected by pre-operative activity levels as measured by the UCLA activity score. There was no significant difference in the mental QOL measure (SF-12 MCS) between the primary and revision THAs. These findings provide a framework for counseling patients undergoing hip revision surgery on predicted post-operative outcomes and PROMs thereby allowing them to be adequately informed about their prognosis.
